# Structures of Gas‐Phase Hydrated Phosphotyrosine Revealed by Soft X‐ray Action Spectroscopy

**DOI:** 10.1002/chem.202403665

**Published:** 2025-01-21

**Authors:** Juliette Leroux, Jean‐Yves Chesnel, Carlos Ortiz‐Mahecha, Aarathi Nair, Bart Oostenrijk, Laura Pille, Florian Trinter, Lucas Schwob, Sadia Bari

**Affiliations:** ^1^ CIMAP CEA/CNRS/ENSICAEN/Université de Caen Normandie 14050 Caen France; ^2^ Deutsches Elektronen-Synchrotron DESY 22603 Hamburg Germany; ^3^ Hamburg University of Technology 21073 Hamburg Germany; ^4^ The Hamburg Centre for Ultrafast Imaging 22761 Hamburg Germany; ^5^ Molecular Physics Fritz-Haber-Institut der Max-Planck-Gesellschaft 14195 Berlin Germany; ^6^ Zernike Institute for Advanced Materials University of Groningen 9747 AG Groningen The Netherlands

**Keywords:** Soft X-ray spectroscopy, Biomolecules, Density functional theory, Nanosolvation

## Abstract

Gas‐phase near‐edge X‐ray absorption mass spectrometry (NEXAMS) was employed at the carbon and oxygen K‐edges to probe the influence of a single water molecule on the protonated phosphotyrosine molecule. The results of the photodissociation experiments revealed that the water molecule forms two bonds, with the phosphate group and another chemical group. By comparing the NEXAMS spectra at the carbon and oxygen K‐edges with density functional theory calculations, we attributed the electronic transitions responsible for the observed resonances, especially the transitions due to the presence of the water molecule. We showed that the water molecule leads to a specific spectral feature in the partial ion yield of hydrated fragments at 536.4 eV. Moreover, comparing the NEXAMS spectra with the calculated structures allowed us to identify three possible structures for singly hydrated phosphotyrosine that agree with the observed fragmentation and resonances.

## Graphical abstract

Gas‐phase near‐edge X‐ray absorption mass spectrometry (NEXAMS) reveals the effects of a single water molecule on protonated phosphotyrosine at the carbon and oxygen K‐edges. The water molecule bonds with the phosphate group, altering the spectral features. Comparisons with density functional theory calculations identify three potential hydrated structures.

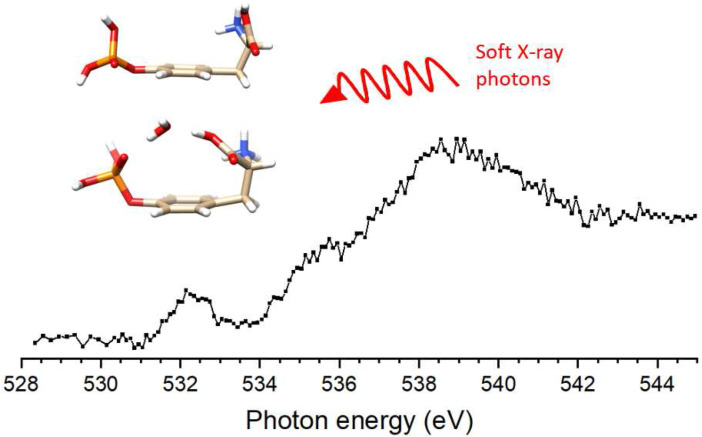

## Introduction

Understanding solvation effects represents a fundamental aspect of chemistry, as the structure and reactivity of molecu‐les in a solvent environment differ significantly from those in vacuum. A single water molecule can already alter the protonation site of a tripeptide.^[1]^ Interactions with solvent molecules can modify the relative stability. It has been shown for a diprotonated organic molecule fully linear in the gas phase that the most stable structure is folded when it interacts with water molecules.^[2]^ The effect of a few water molecules on the structure and the reaction dynamics of amino acids has been studied theoretically,[^3^, ^4^, ^5^] as well as experimentally using mass spectrometry combined with infrared (IR) and ultra‐violet (UV) spectroscopy.[^6^, ^7^, ^8^]

Additionally, soft X‐ray light can be employed to understand the influence of solvents on the properties of biomolecules. Over the last decades, soft X‐ray spectroscopy has become a valuable tool to study the electronic structures of biomolecular systems in the condensed[^9^, ^10^] and gas phases.[^11^, ^12^] Soft X‐rays are element‐specific, allowing site‐selective excitation of a molecule to unoccupied molecular orbitals by tuning the photon energy to a resonance.^[13]^ Recently, Milosavljević et al. performed oxygen K‐shell spectroscopy of an isolated protonated peptide (substance P) solvated with four and eleven water molecules, formed by means of an electrospray ionization source (ESI).^[14]^ By scanning resonant absorption of X‐rays at the core level of the oxygen atoms of the water molecules, they showed that the peptide backbone fragmentation increases compared to the isolated one. This suggests a charge or energy transfer from the solvent to the peptide, such as in intermolecular Coulombic decay (ICD).^[15]^ However, Milosavljević et al. also studied the effect of three water molecules on the stabilization of a peptide dimer upon VUV irradiation and demonstrated that three water molecules already stabilize the dimer by about 1.5 eV and, thereby, prevent damage by the VUV photons.^[16]^ This bimodal behavior remains a puzzle, as the mechanisms behind the nanosolvation effect on photostability are not yet well understood.

**Figure 1:**
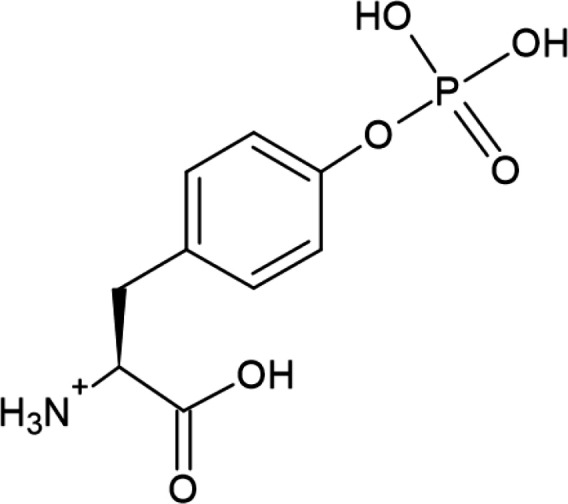
Chemical structure of protonated phosphotyrosine (pTyr).

The phosphorylation of the alcohol group on the side chain of tyrosine, serine, or threonine is one of the most frequent post‐translational modifications (PTMs) of proteins.^[17]^ The replacement of the ‐OH group by a phosphate group $H_{3}{\mathrm{PO}}_{4}$ generally modifies the protein's biological functions. While Milosavljević et al. focused on peptides consisting of natural amino acids,[^14^, ^16^] it is also important to understand the influence of the solvent on the structure and fragmentation process of phosphorylated amino acids and proteins. To understand the effect of a single solvent molecule, we studied protonated phosphotyrosine (hereafter written as pTyr; its chemical structure is shown in Figure 1) in the gas phase as a model for phosphorylation. The presence of the phosphate group is likely to direct water attachment toward it.^[18]^ pTyr has already been studied by infrared multiple photon dissociation (IRMPD) spectroscopy by Scuderi et al.^[19]^ They have shown that adding a water molecule to pTyr redshifts the ammonium NH stretches and the phosphate P=O stretch, which they could link to the formation of a strong hydrogen bond bridge by water between the phosphate group and the ammonium.

In this paper, we extend this work by studying the influence of a single water molecule on the X‐ray fragmentation and electronic structure of pTyr by analysis of the soft X‐ray spectra at the carbon and oxygen K‐edges, aiming at deciphering the location of the water molecule in hydrated pTyr and its impact on the measured spectra. This paper is divided as follows: first, the experimental mass spectra and X‐ray absorption spectra at the carbon and oxygen K‐edges are described. In the last section, a discussion of the potential structural candidates is done in light of the comparison of our experimental and simulation results.

## Results and Discussion

### Mass Spectra

In this section, the ESI‐only spectrum (no photons) of $[\mathrm{pTyr}+H+H_{2}{O]}^{+}$ as well as the photodissociation spectrum of $[\mathrm{pTyr}+H+H_{2}{O]}^{+}$ at the O K‐edge (536.3 eV) will be discussed.

**Figure 2:**
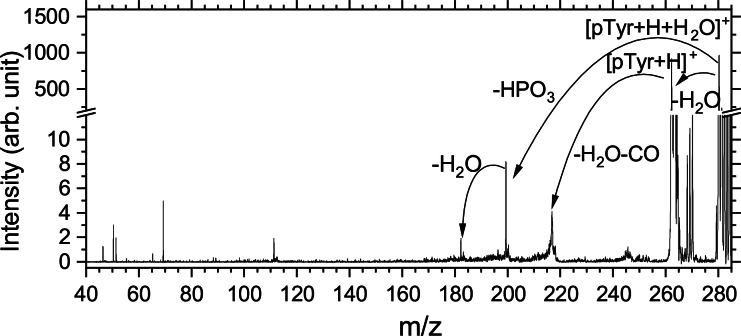
ESI‐only mass spectrum of hydrated phosphotyrosine. The precursor ion $[\mathrm{pTyr}+H+H_{2}{O]}^{+}$ is observed at *m/z* 280.

The ESI‐only spectrum, recorded without photons, is the result of the collisional activation of the molecule with the helium buffer gas. In the ESI‐only mass spectrum reported in Figure 2, we observed multiple neutral losses from hydrated pTyr. The main neutral loss observed is the loss of $H_{2}O$ from the selected parent ion, leading to bare pTyr. Although only the hydrated phosphotyrosine was mass selected in the quadrupole mass spectrometer (QMS), the intensity of bare pTyr was almost equal to that of hydrated pTyr due to collision with the helium buffer gas in the trap. Moreover, bare pTyr could further fragment in the trap before photon irradiation. A corresponding fragment from bare pTyr at *m/z* 216 results from the loss of $H_{2}O$ and CO as already observed by Scuderi et al.^[19]^ Interestingly, this sequential loss of $H_{2}O$ and CO from the carboxyl group is not observed in the case of hydrated pTyr (it would appear at *m/z* 234), indicating that the addition of a water molecule non‐covalently bound to the amino acid affects the possible fragmentation channels.

From hydrated pTyr, we observed the loss of ${\mathrm{HPO}}_{3}$ at *m/z* 200 and the loss of [${\mathrm{HPO}}_{3}+H_{2}O$ ] at *m/z* 182. The loss of ${\mathrm{HPO}}_{3}$ is a characteristic neutral loss in phosphorylated amino acids and peptides.[^20^, ^21^] The process for the loss of [${\mathrm{HPO}}_{3}+H_{2}O$ ] is however unclear. Since the intensity of this peak is much weaker than the ${\mathrm{HPO}}_{3}$ loss for hydrated pTyr, it suggests that either water is subsequently lost after ${\mathrm{HPO}}_{3}$ and bare pTyr does not dissociate via ${\mathrm{HPO}}_{3}$ loss, or that the loss of ${\mathrm{HPO}}_{3}$ from bare pTyr is somehow quenched in comparison to hydrated pTyr. That could be explained if ${\mathrm{HPO}}_{3}$ loss would be a high‐energy dissociation channel, and that the prior loss of water to produce bare pTyr leaves it with much less kinetic energy to initiate an energetic collision with the buffer gas than hydrated pTyr. Our present data does not allow to conclude which of the two processes is here at play.

**Figure 3:**
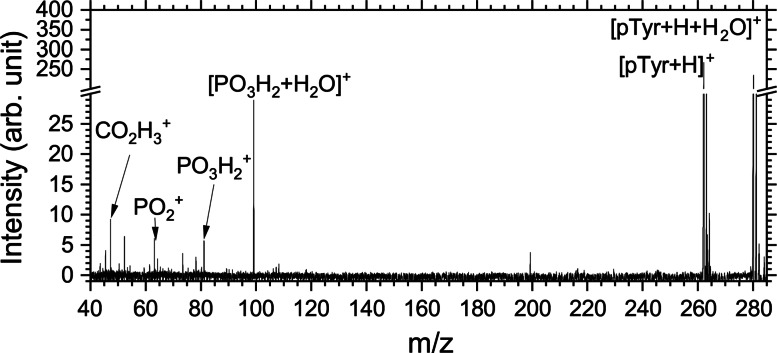
Photodissociation mass spectrum of the mixture of hydrated and bare pTyr measured at 536.3 eV photon energy.

The mass spectrum revealed peaks in the low‐mass region (*m/z* 45 to *m/z* 115) that could not be assigned to fragments of pTyr or its hydrated forms but could be distinguished from pTyr because they did not match the expected fragmentation pattern of pTyr.[^22^, ^23^] We typically expect benzene fragments from the tyrosine side‐chain^[22]^ and phosphate losses.^[23]^ Figure 3 shows the spectra of bare and hydrated pTyr irradiated at 536.3 eV after ESI‐only and photon‐only spectra subtraction.

We observed 13 fragments in total from isolated and hydrated pTyr. A table summarizing the attribution of the different fragments observed after photon irradiation is shown in the SI. Most fragments could be attributed to tyrosine side‐chain fragments such as *m/z* 52, 65, 78, 80, 107, and 108. Fragments at *m/z* 63, 81, and 99 are attributed to fragments of the phosphate group, ${\mathrm{PO}}_{2}^{+}$ , ${\mathrm{PO}}_{3}H_{2}^{+}$ , and ${[\mathrm{PO}}_{3}H_{2}+H_{2}{O]}^{+}$ , while *m/z* 47 and 62 are backbone fragments, respectively, ${\mathrm{CO}}_{2}H_{3}^{+}$ and $C_{2}O_{2}H_{6}^{+}$ . Based solely on this mass spectrum, it is not possible to conclude whether ${\mathrm{PO}}_{3}H_{2}^{+}$ is produced by the evaporation of the water molecule from the hydrated fragment ${[\mathrm{PO}}_{3}H_{2}+H_{2}{O]}^{+}$ or directly from fragmentation of the isolated $[\mathrm{pTyr}+{H]}^{+}$ . This will be further discussed in the context of the partial yield spectra at the O K‐edge (see next section). The observation of the hydrated fragment ${[\mathrm{PO}}_{3}H_{2}+H_{2}{O]}^{+}$ suggests that the non‐covalent bond between pTyr and the water molecule can be conserved upon photoexcitation at 536 eV. This is surprising considering the large internal energy that the system possesses following the photoabsorption. At these photon energies, core photo‐excitation is typically followed by the emission of an Auger‐Meitner electron, which on average leaves behind an excitation energy of 10–20 eV.[^24^, ^25^] This is different from collision‐induced dissociation where, for the deprotonated nucleotide AMP, it has been shown that the hydrated molecule first undergoes loss of the water molecule and then further fragments.^[26]^ In contrast, hydrated fragments have been observed after irradiation at the oxygen K‐edge of a hydrated peptide with four water molecules.^[14]^ This suggests that the fragmentation leading to the ${[\mathrm{PO}}_{3}H_{2}+H_{2}{O]}^{+}$ cation might take place before the intramolecular vibrational redistribution of the internal energy.

It is also worth mentioning that the complementary fragment of ${[\mathrm{PO}}_{3}H_{2}+H_{2}{O]}^{+}$ is the protonated tyrosine amino acid minus one hydrogen atom, that should appear at *m/z* 181. This fragment was absent from our mass spectra, suggesting that the amino acid undergoes further dissociation after the loss of the ${[\mathrm{PO}}_{3}H_{2}+H_{2}{O]}^{+}$ fragment. All fragments mentioned in this section have been used to obtain the near‐edge X‐ray absorption mass spectroscopy (NEXAMS, see sections Methods and SI 1.1) spectra shown in the next section.

To conclude, the two mass spectra described above allow us to make already some assumptions concerning the structure of hydrated pTyr and, more specifically, the binding of the water molecule. On the one hand, the observation of the hydrated fragment ${[\mathrm{PO}}_{3}H_{2}+H_{2}{O]}^{+}$ , after photoabsorption at the O K‐edge, suggests that the water molecule binds to the phosphate group in phosphotyrosine. On the other hand, the fact that, upon collisional activation, the phosphate group ${(\mathrm{HPO}}_{3})$ is lost from hydrated pTyr while the non‐covalently bonded water molecule is retained suggests that the water is not only bound to the phosphate group but must have another established bond with another chemical group of the molecule. In the following, we will show that the examination of the NEXAMS spectra and the comparison with density functional theory (DFT) calculations will allow us to conclude the structure of hydrated pTyr.

## Experimental NEXAMS Spectra

**Figure 4:**
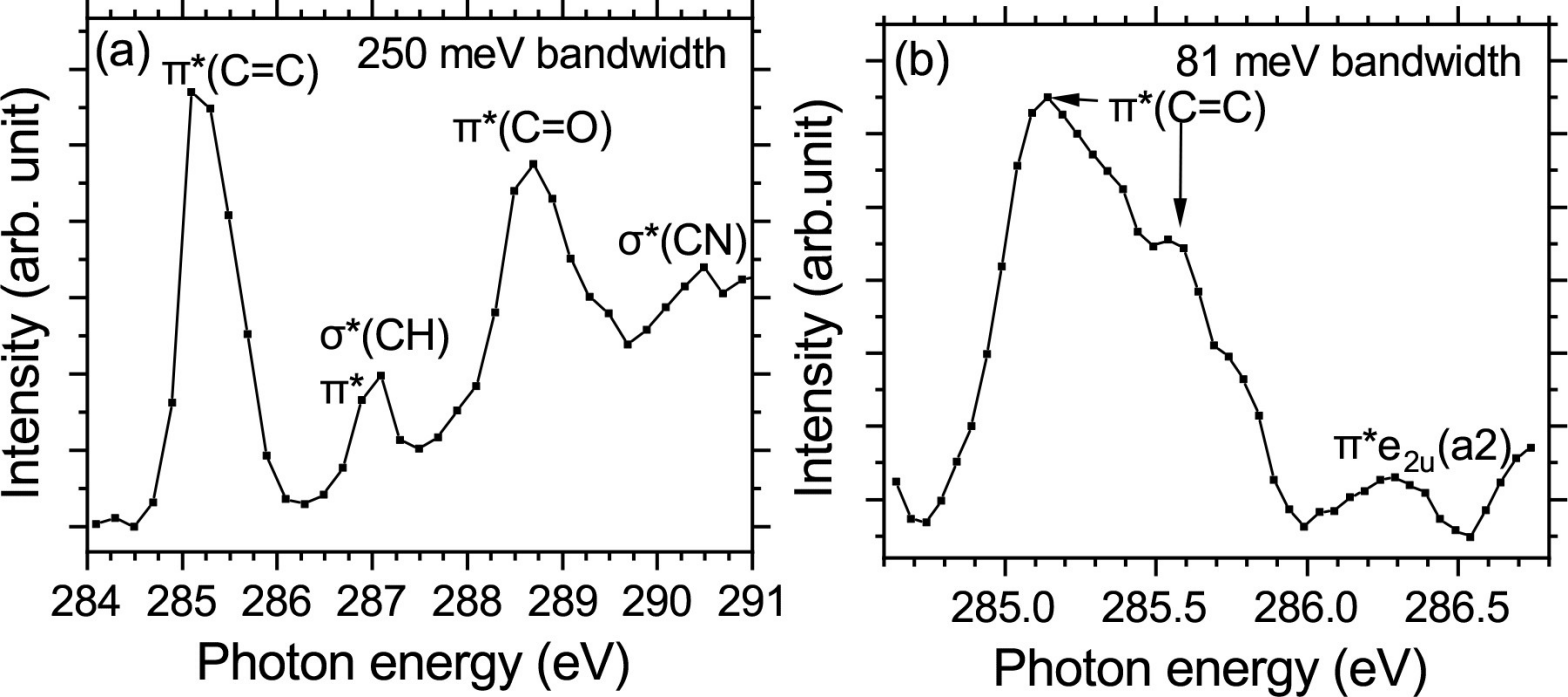
NEXAMS spectra at the C K‐edge of the mixture of hydrated and bare pTyr. In panel (a), the full‐range measurement and in panel (b), the high‐resolution NEXAMS spectrum are shown.

The NEXAMS spectra at the C K‐edge of the mixture of bare and hydrated pTyr are shown in Figure 4. At the C K‐edge, we observed three main resonances, as can be seen in Figure 4(a): the first one is at about 285 eV and originates from a C 1s → π *(C=C) transition from the benzene ring. We observed in the high‐resolution scan around 285 eV that the π *(C=C) resonance is split into two resonances separated by 0.4 eV [see Figure 4(b)]. This is explained by the fact that the molecular environment is not identical for the different carbon atoms of the ring, as has been described in the X‐ray absorption spectrum of neutral benzene and gas‐phase neutral tyrosine.[^27^, ^28^] A second, weaker resonance can be observed in Figure 4(b), located at 286.2 eV. Theoretical and experimental studies have attributed this weak resonance to a dipole‐forbidden C 1s → 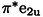 (a_2_) resonance, which is vibronically allowed due to symmetry breaking of the benzene ring.[^28^, ^29^, ^30^]

The second resonance present in Figure 4(a), at about 287 eV, is a combination of two different transitions located at 287.1 eV and 287.4 eV due to $C_{\mathrm{ring}}$ 1s → π * and $C_{\mathrm{ring}}$ 1s → σ *(CH), respectively. This resonance has been observed for solid‐state tyrosine and tyrosine‐containing peptides.[^12^, ^31^, ^32^] The last main resonance at 288.7 eV has been attributed to the C 1s → π * transition from the benzene ring,[^27^, ^28^] more precisely to C 1s → π *(C=O) transitions in small peptides in solid phase[^33^, ^34^] and gas phase.[^12^, ^35^] Accordingly, our calculations indicated that the resonance at 288.7 eV predominantly originates from C 1s → π *(C=O) transitions from the carboxyl group (as explained later). A very weak resonance is observed in the NEXAMS spectrum (Figure 4) and for most partial ion yields (PIYs) (Figure S4) around 290.5 eV. This resonance has already been observed for phenylalanine and tyrosine and is attributed to C 1s → σ *(CC) and C 1s → σ *(CN).^[27]^

All transitions in the NEXAMS spectrum at the C K‐edge can be attributed to transitions already observed in gas‐phase neutral tyrosine or protonated peptides. This could be expected, as phosphorylation of the side chain of tyrosine does not change the direct neighboring atom of the different carbon atoms.

Comparing the partial ion yield spectra of the different fragments in Figure S4, it appeared that the water molecule has little influence at the carbon K‐edge. Even the PIY of the hydrated fragment ${[\mathrm{PO}}_{3}H_{2}+H_{2}{O]}^{+}$ , that undoubtedly originates from $[\mathrm{pTyr}+H+H_{2}{O]}^{+}$ , does not show any clear difference (new peaks or shifts) that could identify an effect of the solvation.

In contrast to the carbon K‐edge, the phosphorylation and the water molecule are anticipated to have significant signatures at the oxygen K‐edge, given their elemental composition. In this energy range, the main resonances of the amino acid are expected at 532.1 eV, 535.2 eV, and 541 eV according to the work of Zhang et al. on neutral tyrosine,^[27]^ whereas for the water molecule, the two main resonances are expected at 534.2 eV and 536.2 eV according to Wilson et al.^[36]^ From the study of Wang et al., a weak resonance due to transitions to the phosphate group is expected around 534.5 eV.^[37]^ However, Ward et al. have shown that the fingerprint of ${\mathrm{PO}}_{4}$ in phosphate‐containing molecules appears as a broad and highly intense resonance at 536.7 eV.^[38]^ Therefore, the oxygen K‐edge allows us to distinguish between the resonances of the different oxygen sites of hydrated pTyr, as we do not expect any overlap between them.

**Figure 5:**
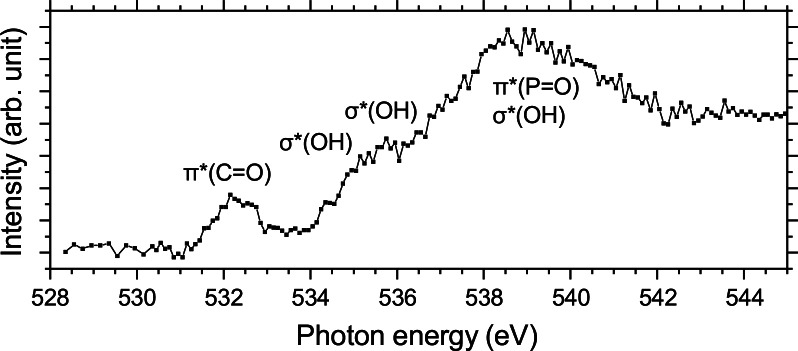
Experimental absorption spectrum of a mixture of $[\mathrm{pTyr}+H+H_{2}{O]}^{+}$ and $[\mathrm{pTyr}+{H]}^{+}$ at the O K‐edge.

In Figure 5, the O K‐edge NEXAMS spectrum of the mixture of $[\mathrm{pTyr}+H+H_{2}{O]}^{+}$ and $[\mathrm{pTyr}+{H]}^{+}$ is shown. We observed one main resonance at 532.2 eV, which was also observed in the X‐ray absorption spectrum of neutral tyrosine.^[27]^ The authors attributed this resonance to an O 1s → π *(C=O) transition from the carboxyl group. Our calculations at the O K‐edge confirmed that this attribution is valid for both bare pTyr and hydrated pTyr (see next section). The weak resonance at 534.5 eV, appearing as a shoulder left of the broad feature, can be attributed to a σ *(OH) transition from the water molecule, as discussed in the following. The resonance near 536 eV stems from transitions from O 1s → σ *(OH) mostly from the carboxyl group of the molecule and with a small contribution from the water molecule when it forms a bridge between the phosphate group and the carboxyl group, according to our calculations (see the OP_bridge structure in the following). The last broad resonance at 539 eV is due to many electronic transitions, mainly π *(P=O) and σ *(OH) transitions from the phosphate group^[38]^ (see the next section for a discussion of the different calculated conformers and specific attributions of the resonances).

**Figure 6:**
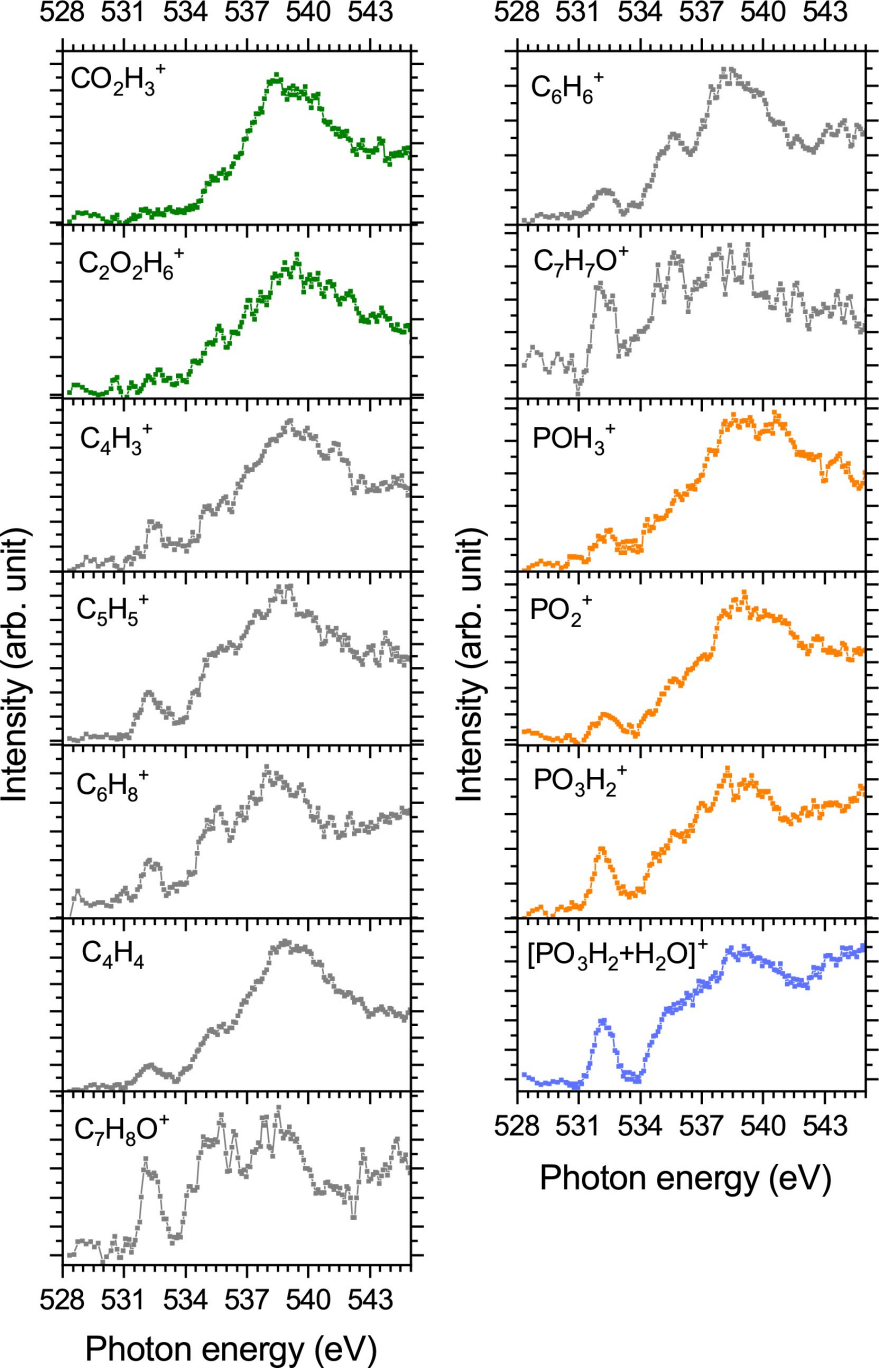
PIYs of the fragments originating from a mixture of $[\mathrm{pTyr}+H+H_{2}{O]}^{+}$ and [pTyr+H]^+^ at the oxygen K‐edge. The PIYs of the carboxyl‐containing fragments are shown in green, and the PIYs of tyrosine side‐chain fragments are shown in grey. The PIYs of phosphorus‐containing fragments are shown in orange, and the PIY of the hydrated fragment is shown in blue.

As the NEXAMS spectrum shown in Figure 5 is the result of the irradiation of both pTyr and hydrated pTyr, a more precise conclusion cannot be made based only on the NEXAMS spectrum. However, additional information can be extracted from the PIYs at the oxygen K‐edge, especially from the PIY of the hydrated fragment ${[\mathrm{PO}}_{3}H_{2}+H_{2}{O]}^{+}$ . In Figure 6, the PIYs of every fragment at the oxygen K‐edge are shown. Some differences in the PIYs of the fragments can be observed. Interestingly, the two backbone fragments ${\mathrm{CO}}_{2}H_{3}^{+}$ and $C_{2}O_{2}H_{6}^{+}$ do not exhibit the first O1s → π *(C=O) resonance at 532.15 eV. These two fragments contain an intact carboxyl group. The absence of the resonance suggests that exciting the π *(C=O) orbitals will first and foremost induce cleavage of the C=O and/or C‐OH bonds in the carboxyl group. In this case, fragments containing an intact COOH group cannot be produced through this resonant excitation.

**Figure 7:**
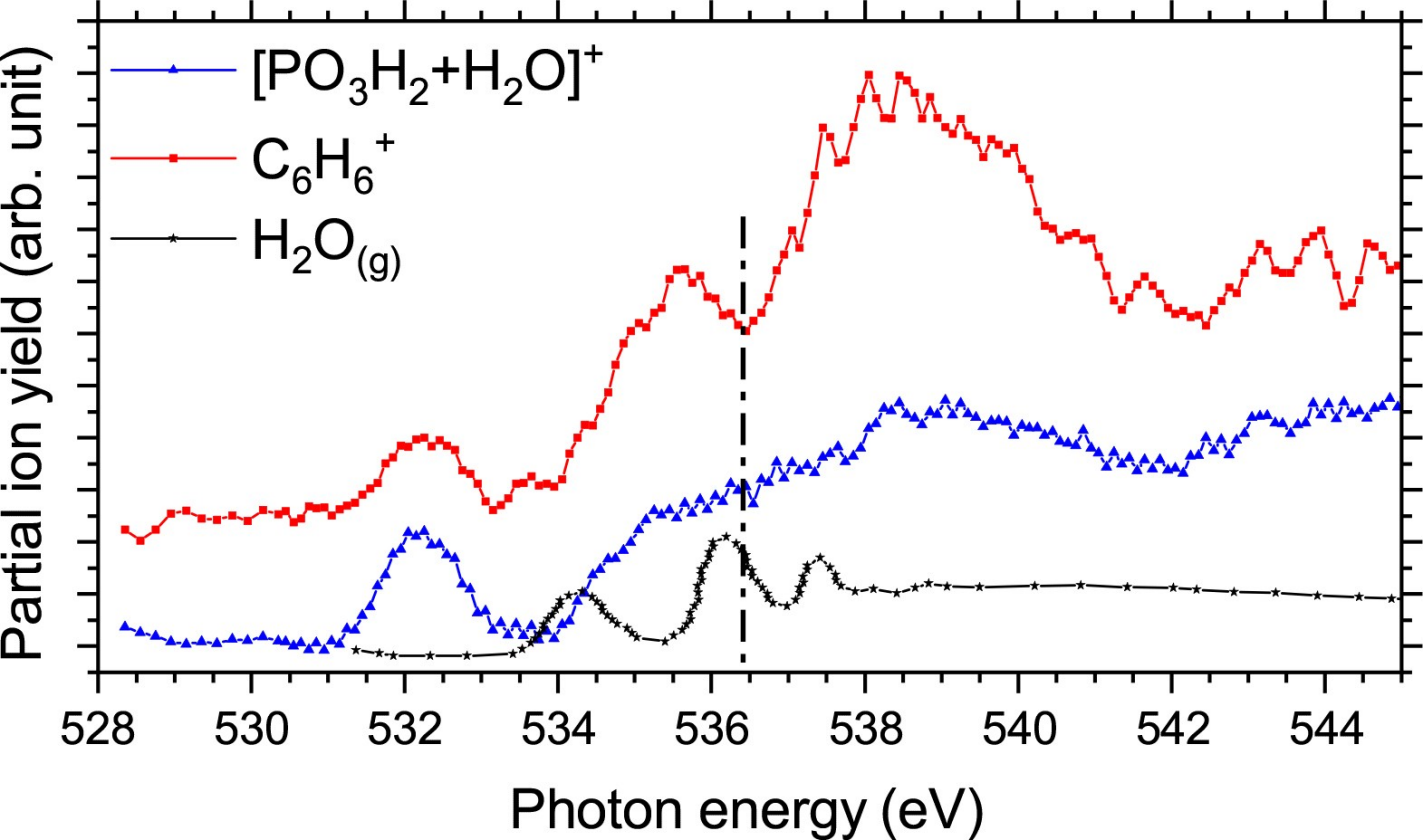
Comparison of the ion yield spectra of $C_{6}H_{6}^{+}$ and ${[\mathrm{PO}}_{3}H_{2}+H_{2}{O]}^{+}$ with the experimental TIY of gaseous water, adapted from Wilson et al.^[36]^ A vertical dashed line has been added at 536.4 eV (resonance of the water molecule^[36]^) to guide the eyes.

We observed that for all tyrosine side‐chain fragments, plotted in grey in Figure 6, as well as for the phosphate‐containing fragments ${\mathrm{PH}}_{3}O^{+}$ and ${\mathrm{PO}}_{2}^{+}$ , the relative intensity between the resonance at 532.15 eV and the resonances at higher photon energies is similar. The fragment ${[\mathrm{PO}}_{3}H_{2}+H_{2}{O]}^{+}$ and its bare form ${\mathrm{PO}}_{3}H_{2}^{+}$ present a different ratio between the resonances at 532.15 eV and at 535 eV and might therefore originate from different processes than the tyrosine side‐chain fragments. This will be discussed further at the end of this section. We observed differences between a tyrosine side‐chain fragment ($C_{6}H_{6}^{+}$ ) coming from the bare pTyr and the hydrated fragment ${[\mathrm{PO}}_{3}H_{2}+H_{2}{O]}^{+}$ . Specifically, we observed for $C_{6}H_{6}^{+}$ a dip at 536.5 eV whereas for ${[\mathrm{PO}}_{3}H_{2}+H_{2}{O]}^{+}$ the resonances at 535.5 eV and the one centered around 539 eV are not well resolved (see Figure 7). This indicates that for ${[\mathrm{PO}}_{3}H_{2}+H_{2}{O]}^{+}$ an additional resonance appears at about 536.4 eV. We explain this new resonance by the presence of transitions from the water molecule around 536 eV, as shown in Figure 7. Wilson et al. measured the total ion yield spectrum of gas‐phase water at the oxygen K‐edge; they observed three main peaks in the spectrum, at 534.2 eV, 536.2 eV, and 537.5 eV^[36]^ assigned to transitions from the ground state to a mixture of empty molecular orbitals and Rydberg states.^[39]^

As mentioned in the previous section, it was not possible to conclude that ${\mathrm{PO}}_{3}H_{2}^{+}$ is produced by the evaporation of the water molecule from ${[\mathrm{PO}}_{3}H_{2}+H_{2}{O]}^{+}$ on the basis of only the photodissociation mass spectrum. However, it is now possible to draw some conclusions by comparing the PIYs of both fragments at the oxygen K‐edge. As illustrated in Figure 7 highlighting the differences between the $C_{6}H_{6}^{+}$ and ${[\mathrm{PO}}_{3}H_{2}+H_{2}{O]}^{+}$ fragments, the shape of the PIY spectra of bare pTyr fragments differ from that of hydrated pTyr fragments. If ${\mathrm{PO}}_{3}H_{2}^{+}$ comes from the evaporation of the water molecule from ${[\mathrm{PO}}_{3}H_{2}+H_{2}{O]}^{+}$ after irradiation with photons, the shape of the PIY spectrum of the hydrated fragment ${[\mathrm{PO}}_{3}H_{2}+H_{2}{O]}^{+}$ should be similar to that of its bare form ${\mathrm{PO}}_{3}H_{2}^{+}$ . Figure S5 (SI) shows that their PIY spectra are identical once normalized on the first resonance at 532.15 eV, thus supporting the hypothesis that, after being produced, the hydrated fragment may have a high internal energy and loses its water molecule. To obtain more insight into the different electronic transitions and the possible structures of hydrated pTyr, DFT calculations have been performed and compared with the X‐ray absorption spectra at the C and O K‐edges. This will be discussed in the next section.

### Comparison of the Calculated Absorption Spectra with the NEXAMS Spectra

The analysis of the photodissociation mass spectra drove all calculations for the hydrated conformers. The spectra revealed that the main hydrated fragment, at *m/z* 99 (${[\mathrm{PO}}_{3}H_{2}+H_{2}{O]}^{+}$ ), contains the phosphate group, thereby directing the structural analysis towards conformations where water binds to the phosphate group. Moreover, the loss of ${\mathrm{HPO}}_{3}$ from hydrated pTyr was observed without the loss of $H_{2}O$ , indicating that the water probably binds not only to the phosphate group.

**Figure 8:**
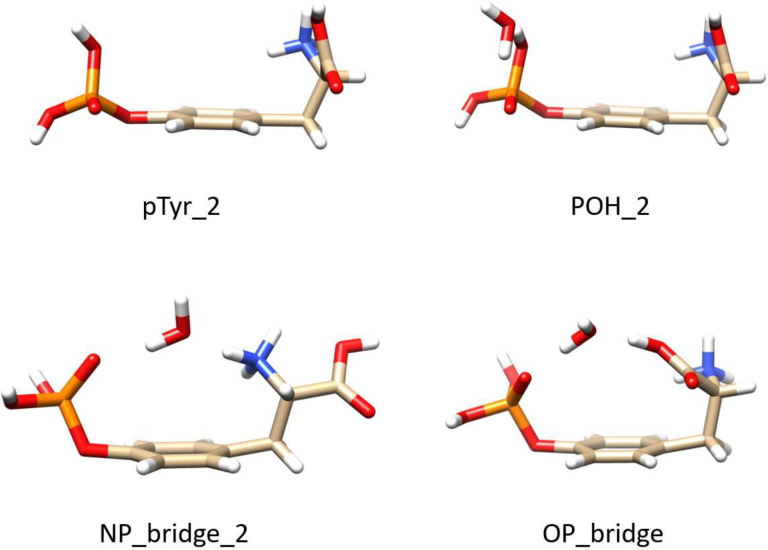
Structures of the bare pTyr and the three singly hydrated conformers of pTyr. POH_2 has the water molecule bound to the phosphate group, while NP_bridge_2 and OP_bridge have the water molecule bound to the phosphate group and the ammonium or carboxyl group, respectively.

The structures of the three hydrated conformers and the bare one that were considered (following the method detailed in the Supporting Information) are shown in Figure 8. The selected isolated pTyr (pTyr_2) has the phosphate and amino‐acid groups lying on the same side of the phenyl plane. From this structure, three singly hydrated pTyr conformers were considered. POH_2 has the same structure as pTyr_2 with the water molecule binding to the phosphate. From the previous work of Scuderi et al.,^[19]^ we could also expect the water molecule to bind only to the ammonium to form a strong hydrogen bond. Such a structure was considered during our structural search but not further considered as its calculated spectra did not agree with our NEXAMS spectra (see the Supporting Information). Moreover, the water molecule may bridge the phosphate group and the ammonium or the carboxyl group, as for the structures NP_bridge_2 and OP_bridge presented in Figure 8.

**Figure 9:**
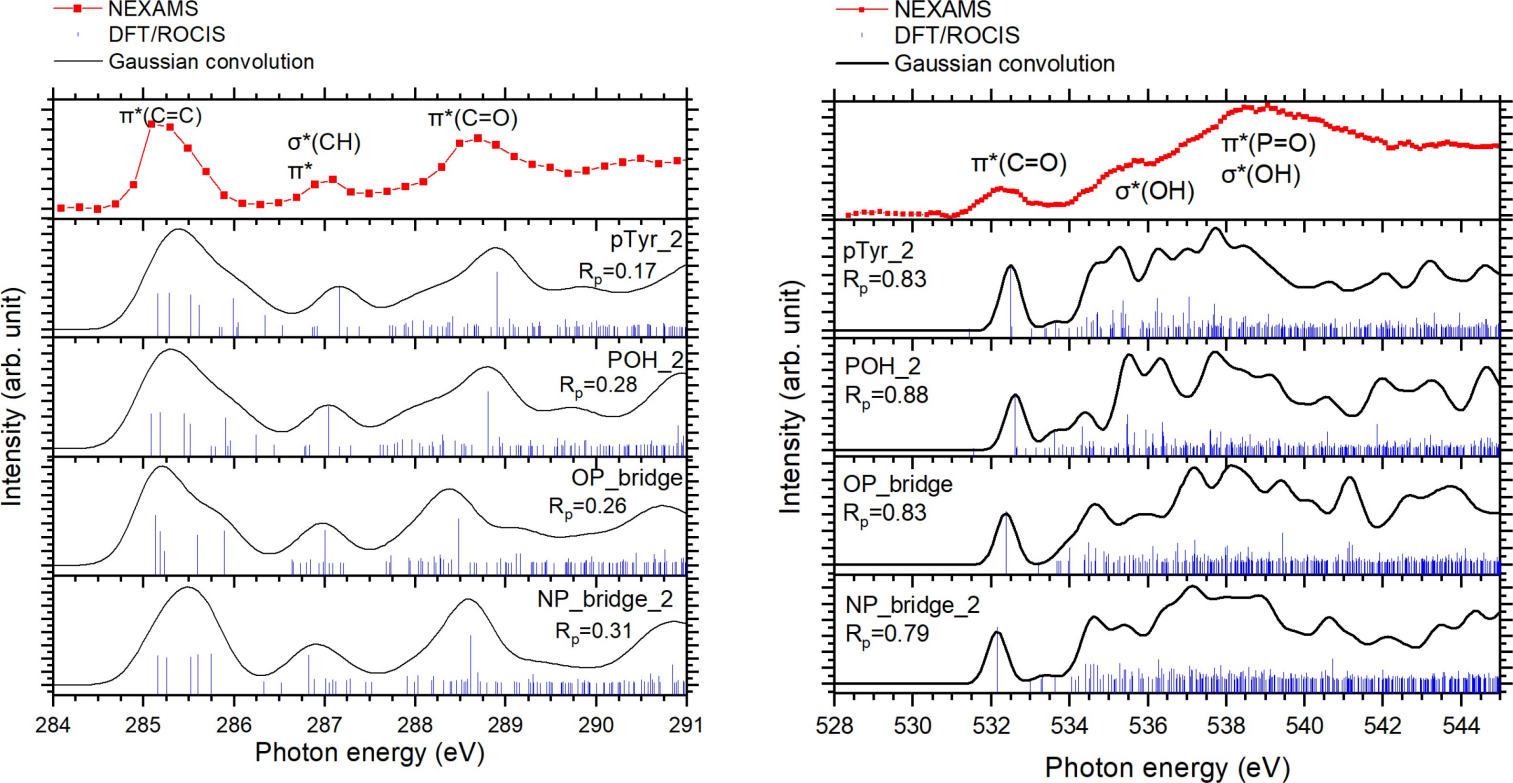
Comparison between the calculated X‐ray absorption spectra of the bare pTyr (pTyr_2) and the three selected hydrated pTyr conformers (POH_2, OP_bridge, and NP_bridge_2) and the experimental NEXAMS spectra (red) at the C K‐edge (left) and at the O K‐edge (right). Their Pendry factor (R_*p*_) is also given.

Figure 9 compares the experimental NEXAMS spectra at the carbon and oxygen K‐edges with the calculated spectra of the four selected conformers. To compare the theoretical spectra with the experimental one, we used the Pendry reliability factor $R_{p}$ for an unbiased criterion.^[40]^ A perfect agreement between the theoretical spectrum and the experimental one is represented by $R_{p}=0$ , while $R_{p}=1$ means no agreement. pTyr_2 is the isolated conformer that was chosen to be the final representative for bare pTyr after a comparison of its calculated spectrum with the NEXAMS spectrum at the carbon K‐edge (see Supporting Information). From the comparison of the NEXAMS spectrum and the calculated spectra at the C K‐edge, we infer that all four conformers are in agreement and can be possible fitting structures for bare and hydrated pTyr in our experimental setup. Their $R_{p}$ factor, at the C K‐edge, demonstrates the good agreement between the experimental spectrum and the calculated ones. However, to confirm that the pTyr_2 conformer and the three hydrated conformers can be good structural candidates for pTyr, their calculated spectra should also be in agreement with the experimental spectrum at the O K‐edge.

The right panel of Figure 9 shows the comparison of the NEXAMS spectra with the calculated spectra at the oxygen K‐edge for the three singly hydrated conformers, POH_2 (where the water binds to the phosphate group), OP_bridge (where the water molecule forms a bridge between the carboxyl group and the phosphate group), and NP_bridge_2 (where the water molecule forms a bridge between the ${\mathrm{NH}}_{3}^{+}$ group and the phosphate group) and the bare pTyr_2. Despite showing Pendry factors $R_{p}$ that are closer to 1, the computed transitions and intensities present a qualitative good agreement with the experiment. Figure S6 highlights the contributions of the oxygen atoms of the water molecule, the phosphate group, and the carboxyl group to the calculated spectra.

At first glance, we observed that the calculated spectra of OP_bridge and NP_bridge_2 are similar in shape. The first resonance at 532.15 eV is entirely due to O 1s → π *(C=O) from the carboxyl group, with no transition from other oxygen atoms in the molecules, and the broad feature between 535 and 540 eV comes mostly from transitions from an oxygen atom of the phosphate group exciting σ * antibonding orbitals. For both conformers bridging the phosphate group and either the N‐terminus or the carboxyl group (labeled NP_bridge_2 and OP_bridge, respectively), the water molecule does not exhibit strong transitions and seems to act more like a spectator in the calculation, except for a weak resonance forming a shoulder at 534 eV on the resonance at 534.7 eV. However, this shoulder is more pronounced for the OP_bridge conformer compared to NP_bridge_2. This weak resonance at 534 eV is due to transitions from the oxygen atom of the water molecule into a σ *(OH) orbital (LUMO+7, Figure S7) where most of the density is located on the water molecule (see Figure S7). This could explain the low‐intensity resonance at 534.2 eV in our experimental spectrum, which would also be consistent with the TIY spectrum of gas‐phase water of Wilson et al.^[36]^ This transition is also observed in the calculated spectrum of NP_bridge_2 at 533.5 eV (LUMO+3, Figure S7), but the intensity of this transition is even lower. Thus, this transition does not contribute significantly to the total calculated spectrum. Moreover, for NP_bridge_2, the resonance at 534.7 eV is shared equally by contributions of the carboxyl and phosphate groups through O 1s → π *(C=O) and O 1s → σ *(OH) transitions (LUMO and LUMO+6, Figure S7). For the OP_bridge conformer, starting from 535 eV, the intense transitions are all due to the phosphate group (LUMO+14, Figure S7).

Furthermore, the POH_2 conformer exhibits a calculated spectrum rather different from the bridge conformers, where the water molecule is responsible for a strong transition at 533.6 eV (LUMO+6, Figure S7) and partly contributes to the resonance at 535.5 eV which is shared with transitions from oxygen atoms of the carboxyl group to the σ *(OH) orbital. From 536 eV to 540 eV, the oxygen atoms of the phosphate group are excited to π *(C=O) and σ *(OH) orbitals (LUMO+1 and LUMO+4, Figure S7).

The differences in the lower energy range of the calculated spectra can be explained by the differences in structure between POH_2 and the two conformers forming a bridge. The resonance around 534 eV (at 533.5 eV for POH_2) is due to transitions from the water molecule into the σ *(OH) orbital, mainly where most of the electronic density is located around the water molecule with a small contribution of the ‐OH group of the phosphate group (LUMO+6, Figure S7). As POH_2 makes only one hydrogen bond with the water molecule, the water molecule interacts less with pTyr compared to the bridge structures where water is more strongly bound to the biomolecule. For the two structures NP_bridge_2 and OP_bridge, the electronic density is shared between the water molecule and pTyr through the phosphate group and either the N‐terminus or the carboxyl group. That is why for OP_bridge and NP_bridge_2, the intensity of these water‐related transitions is very low, and most of the electronic transitions in the calculated spectra of both conformers come from electronic excitation from the carboxyl and phosphate groups. Therefore, the calculated oxygen K‐edge spectra for both bridge conformers resemble more the calculated spectrum of pTyr_2 (see Figure 9).

Moreover, from the NEXAMS spectra, it is clear that no intense transitions are present between the O 1s → π *(C=O) transitions at 532.15 eV and the O 1s → σ *(OH) transitions at 535.5 eV, even though the possibility that some transitions are present cannot be ruled out (the TIY intensity being non‐zero between the two resonances). Thus, we could conclude that POH_2 can be present in our experimental setup but is not the major conformer of the target ion cloud. However, from the ESI‐only and the photodissociation mass spectra, we concluded that the water molecule not only binds to the phosphate group but should bind to another group to allow the loss of ${\mathrm{HPO}}_{3}$ without the loss of the water molecule. Therefore, the two bridge conformers are good structural candidates. Of both conformers, OP_bridge shows a stronger water transition at 534 eV, which might explain our experimental resonance at 534.3 eV.

Scuderi et al. showed that for singly hydrated protonated phosphotyrosine, the most stable conformers have the water molecule bridging the phosphate and the ammonium, where the water is an acceptor from the N−H and a donor to O=P.^[19]^ This is similar to what we obtained for the NP_bridge_2 and OP_bridge structures. For the OP_bridge structure, $H_{2}O$ is an acceptor from the ‐OH of the carboxyl group and a donor to O=P. These authors also found a similar structure to OP_bridge during their structural search, but they did not consider it further.^[19]^ We conclude that, even if we cannot rule out the possibility of POH_2 or NP_bridge_2 and similar structures, OP_bridge best agrees with the NEXAMS spectra at the carbon and oxygen K‐edges. The OP_bridge structure is therefore chosen as the structural representative of hydrated pTyr in our experimental conditions.

## Conclusions

In conclusion, we have shown that the combination of DFT/ROCIS calculations and soft X‐ray action spectroscopy allows the determination of the location of the water molecule. We have measured the experimental NEXAMS spectra at the carbon and oxygen K‐edges of a mixture of isolated and singly hydrated protonated phosphotyrosine to investigate the effect of a single water molecule on the fragmentation and to search for the spectroscopic fingerprints of the biomolecule in the soft X‐ray range. The interpretation of these experimental spectra could be done by comparison with DFT/ROCIS calculations. Following an initial structural search, three possible conformers were calculated based on the insights given by the dissociation mass spectra: one in which the water molecule binds exclusively to the phosphate group and two others where the water molecule forms a bridge between the phosphate group and either the carboxyl group or the N‐terminus. The comparison of the NEXAMS spectra with the calculated spectra of these three conformers at the oxygen K‐edge allowed us to rule out the first conformer. Based on the photodissociation mass spectrum and the comparison between the calculated spectra and NEXAMS spectra, we concluded that the most representative conformer under our experimental conditions was a structure where the water molecule forms a bridge between the phosphate and the carboxyl groups. Additionally, since the NEXAMS technique is element‐specific, the water molecule could be directly probed at the oxygen K‐edge. We established at the O K‐edge that the PIYs of a fragment coming from the bare pTyr have an electronic signature different from that of the hydrated fragment ${[\mathrm{PO}}_{3}H_{2}+H_{2}{O]}^{+}$ , which shows an additional transition at 536.4 eV from the water molecule. Finally, our experimental technique needs to be improved to probe the bare and hydrated molecule separately, for example, using resonant extraction^[41]^ in the trap, which would allow selective removal of one species from the trap or a dedicated electrospray source that would enhance the yield of hydrated species.

### Methods

Phosphotyrosine ($C_{9}H_{12}{\mathrm{NO}}_{6}P$ ) was purchased from Sigma‐Aldrich at ≥95 % purity and used without further purification. The sample solution was prepared at 1.5 mM concentration in 1 : 1 water/methanol with 1 % of formic acid to ensure the protonation.

The results were obtained during an experimental campaign by coupling our home‐built tandem mass spectrometer to the P04 soft X‐ray beamline at the PETRA III synchrotron (DESY, Hamburg, Germany). More details on the experimental method can be found in the Supporting Information.

Soft X‐ray action spectroscopy was employed to obtain the total ion yield (TIY) spectra, the so‐called NEXAMS spectra, of pTyr at the C and O K‐edges. This was achieved by summing the peak areas of all detected fragments of bare and hydrated pTyr in the photoinduced mass spectra across the scanned photon‐energy range. The NEXAMS spectra can be assumed to be good approximations of the X‐ray absorption cross section and, thus, comparable to the calculated oscillator strengths. The partial ion yields for individual fragments are the results of the convolution of the absorption cross section of the molecule with the branching ratio of the different fragmentation pathways. By summing up the contributions of all fragments into a total ion yield spectrum, we approach the result of an absorption spectrum.^[42]^

To assign the different resonances of the NEXAMS spectra at the C and O K‐edges, theoretical calculations were performed to compute the X‐ray absorption spectra (XAS) of structural candidates for pTyr and hydrated pTyr. All calculations were performed using the combination of the density functional theory (DFT) and the restricted open‐shell configuration interaction with singles (ROCIS) method, employing the TZVP Ahlrichs basis set^[43]^ in combination with the B3LYP exchange‐correlation functional.^[44]^ Relativistic effects were considered using the zeroth order regular approximation (ZORA).^[45]^ All *ab initio* calculations have been performed using the ORCA electronic‐structure package.^[46]^ The complete procedure for finding isolated and singly hydrated structural candidates for phosphotyrosine is explained in the Supporting Information.

To compare with the experimental results, the calculated X‐ray absorption spectra were broadened using a Gaussian profile with a full width at half maximum (FWHM) of 0.6 eV to account for the experimental photon‐energy resolution, the core‐hole lifetime, and the electronic broadening of the calculated transitions.[^47^, ^48^] The energy shift of the theoretical spectra has been defined by matching the first resonance of the theoretical spectra to the corresponding one in the experimental spectra, representing the 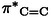 resonance in the case of the C K‐edge calculated spectra and the 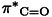 resonance in the case of the O K‐edge calculated spectra.^[49]^ This gives a shift of around 11.5 eV in the case of the C K‐edge and 14 eV in the case of the O K‐edge calculations.

## Conflict of Interests

The authors declare no conflict of interest.

1

## Supporting information

As a service to our authors and readers, this journal provides supporting information supplied by the authors. Such materials are peer reviewed and may be re‐organized for online delivery, but are not copy‐edited or typeset. Technical support issues arising from supporting information (other than missing files) should be addressed to the authors.

Supporting Information

## Data Availability

The data that support the findings of this study are available from the corresponding author upon reasonable request.

## References

[1] J. M. Voss , K. C. Fischer , E. Garand , J. Mol. Spectrosc. 2018, 347, 28.

[2] M. Demireva , J. T. O'Brien , E. R. Williams , J. Am. Chem. Soc. 2012, 134, 11216. 22708846 10.1021/ja303313p

[3] C. M. Aikens , M. S. Gordon , J. Am. Chem. Soc. 2006, 128, 12835. 17002379 10.1021/ja062842p

[4] D. S. Ahn , S. W. Park , I. S. Jeon , M. K. Lee , N. H. Kim , Y. H. Han , S. Lee , J. Phys. Chem. B 2003, 107, 14109.

[5] L. C. Snoek, R. T. Kroemer, J. P. Simons, *Phys. Chem. Chem. Phys*., **2002**, *4*, 2130, DOI: 10.1039/B200059H.

[6] K. C. Fischer , S. L. Sherman , E. Garand , J. Phys. Chem. A 2020, 124, 1593. 32030984 10.1021/acs.jpca.9b11977

[7] K. C. Fischer , S. L. Sherman , J. M. Voss , J. Zhou , E. Garand , J. Phys. Chem. A 2019, 123, 3355. 30908047 10.1021/acs.jpca.9b01578

[8] P. Çarçabal , R. T. Kroemer , L. C. Snoek , J. P. Simons , J. M. Bakker , I. Compagnon , G. Meijer , G. von Helden , Phys. Chem. Chem. Phys. 2004, 6, 4546, DOI: 10.1039/B411757C.

[9] P. Leinweber , J. Kruse , F. L. Walley , A. Gillespie , K. U. Eckhardt , R. Blyth , T. Regier , J. Synchrotron Radiat. 2007, 14, 500. 17960033 10.1107/S0909049507042513

[10] E. Otero , S. G. Urquhart , J. Phys. Chem. A 2006, 110, 12121. 17078606 10.1021/jp064082a

[11] S. Bari , D. Egorov , T. L. C. Jansen , R. Boll , R. Hoekstra , S. Techert , V. Zamudio-Bayer , C. Bülow , R. Lindblad , G. Leistner , A. Lawicki , K. Hirsch , P. S. Miedema , B. von Issendorff , J. T. Lau , T. Schlathölter , Chem. Eur. J. 2018, 24, 7631. 29637635 10.1002/chem.201801440 PMC6001477

[12] S. Dörner , L. Schwob , K. Atak , K. Schubert , R. Boll , T. Schlathölter , M. Timm , C. Bülow , V. Zamudio-Bayer , B. von Issendorff , J. T. Lau , S. Techert , S. Bari , J. Am. Soc. Mass Spectrom. 2021, 32, 670. 33573373 10.1021/jasms.0c00390

[13] G. Hähner , Chem. Soc. Rev. 2006, 35, 1244. 17225886 10.1039/b509853j

[14] A. R. Milosavljević , K. Jänkälä , M. L. Ranković , F. Canon , J. Bozek , C. Nicolas , A. Giuliani , Phys. Chem. Chem. Phys. 2020, 22, 12909, DOI: 10.1039/D0CP00994F. 32347253

[15] T. Jahnke , U. Hergenhahn , B. Winter , R. Dörner , U. Frühling , P. V. Demekhin , K. Gokhberg , L. S. Cederbaum , A. Ehresmann , A. Knie , A. Dreuw , Chem. Rev. 2020, 120, 11295. 33035051 10.1021/acs.chemrev.0c00106 PMC7596762

[16] A. R. Milosavljevic , V. Z. Cerovski , F. Canon , L. Nahon , A. Giuliani , Angew. Chem. Int. Ed. 2013, 52, 7286, DOI: 10.1002/anie.201301667. 23744727

[17] S. Ramazi , J. Zahiri , Database 2021, 2021, 1, DOI: 10.1093/database/baab012. PMC8040245 33826699

[18] F. Calvo , J. Douady , Phys. Chem. Chem. Phys. 2010, 12, 3404. 20336245 10.1039/b923972c

[19] D. Scuderi , J. M. Bakker , S. Durand , P. Maitre , A. Sharma , J. K. Martens , E. Nicol , C. Clavaguéra , G. Ohanessian , Int. J. Mass Spectrom. 2011, 308, 338.

[20] C. F. Correia , P. O. Balaj , D. Scuderi , P. Maitre , G. Ohanessian , J. Am. Chem. Soc. 2008, 130, 3359. 18293967 10.1021/ja073868z

[21] P. J. Boersema , S. Mohammed , A. J. R. Heck , J. Mass Spectrom. 2009, 44, 861. 19504542 10.1002/jms.1599

[22] P. Zhang , W. Chan , I. L. Ang , R. Wei , M. M. T. Lam , K. M. K. Lei , T. C. W. Poon , Sci. Rep. 2019, 9, 6453, DOI: 10.1038/s41598-019-42777-8. 31015571 PMC6478932

[23] J. Li , X. Zhan , Mass Spectrom. Rev. 2023, 43, 857, DOI: 10.1002/mas.21836. 36789499

[24] D. Egorov , L. Schwob , M. Lalande , R. Hoekstra , T. Schlathölter , Phys. Chem. Chem. Phys. 2016, 18, 26213. 27722598 10.1039/c6cp05254a

[25] A. Sanchez-Gonzalez , T. R. Barillot , R. J. Squibb , P. Kolorenč , M. Agaker , V. Averbukh , M. J. Bearpark , C. Bostedt , J. D. Bozek , S. Bruce , S. C. Montero , R. N. Coffee , B. Cooper , J. P. Cryan , M. Dong , J. H. D. Eland , L. Fang , H. Fukuzawa , M. Guehr , M. Ilchen , A. S. Johnsson , C. Liekhus-S , A. Marinelli , T. Maxwell , K. Motomura , M. Mucke , A. Natan , T. Osipov , C. Östlin , M. Pernpointner , V. S. Petrovic , M. A. Robb , C. Sathe , E. R. Simpson , J. G. Underwood , M. Vacher , D. J. Walke , T. J. A. Wolf , V. Zhaunerchyk , J. E. Rubensson , N. Berrah , P. H. Bucksbaum , K. Ueda , R. Feifel , L. J. Frasinski , J. P. Marangos , J. Phys. B 2015, 48, DOI: 10.1088/0953-4075/48/23/234004.

[26] B. Liu , S. B. Nielsen , P. Hvelplund , H. Zettergren , H. Cederquist , B. Manil , B. A. Huber , Phys. Rev. Lett. 2006, 97, 133401. 17026030 10.1103/PhysRevLett.97.133401

[27] W. Zhang , V. Carravetta , O. Plekan , V. Feyer , R. Richter , M. Coreno , K. C. Prince , J. Chem. Phys. 2009, 131. 10.1063/1.3168393 19624235

[28] R. Püttner , C. Kolczewski , M. Martins , A. S. Schlachter , G. Snell , M. Sant'Anna , J. Viefhaus , K. Hermann , G. Kaindl , Chem. Phys. Lett. 2004, 393, 361. 10.1063/1.2139674 16438578

[29] W. H. Schwarz , T. C. Chang , U. Seeger , K. H. Hwang , Chem. Phys. 1987, 117, 73.

[30] E. E. Rennie , B. Kempgens , H. M. Köppe , U. Hergenhahn , J. Feldhaus , B. S. Itchkawitz , A. L. D. Kilcoyne , A. Kivimäki , K. Maier , M. N. Piancastelli , M. Polcik , A. Rüdel , A. M. Bradshaw , J. Chem. Phys. 2000, 113, 7362.

[31] J. Boese , A. Osanna , C. Jacobsen , J. Kirz , J. Electron Spectrosc. Relat. Phenom. 1997, 85, 9.

[32] R. M. Petoral Jr. , K. Uvdal , J. Electron Spectrosc. Relat. Phenom. 2003, 128, 159.

[33] M. L. Gordon , G. Cooper , C. Morin , T. Araki , C. C. Turci , K. Kaznatcheev , A. P. Hitchcock , J. Phys. Chem. A 2003, 107, 6144.

[34] Y. Zubavichus , A. Shaporenko , M. Grunze , M. Zharnikov , J. Phys. Chem. A 2005, 109, 6998. 16834062 10.1021/jp0535846

[35] A. R. Milosavljević , C. Nicolas , M. L. Ranković , F. Canon , C. Miron , A. Giuliani , J. Phys. Chem. Lett. 2015, 6, 3132, DOI: 10.1021/acs.jpclett.5b01288.

[36] K. R. Wilson , B. S. Rude , T. Catalano , R. D. Schaller , J. G. Tobin , D. T. Co , R. J. Saykally , J. Phys. Chem. B 2001, 105, 3346.

[37] X. Wang , S. Rathnachalam , V. Zamudio-Bayer , K. Bijlsma , W. Li , R. Hoekstra , M. Kubin , M. Timm , B. von Issendorff , J. T. Lau , S. Faraji , T. Schlathölter , Phys. Chem. Chem. Phys. 2022, 24, 7815. 35297440 10.1039/d1cp05741c PMC8966622

[38] J. D. Ward , M. Bowden , C. T. Resch , S. Smith , B. K. McNamara , E. C. Buck , G. C. Eiden , A. M. Duffin , Geostand. Geoanal. Res. 2016, 40, 135.

[39] M. N. Piancastelli , A. Hempelmann , F. Heiser , O. Gessner , A. Rüdel , U. Becker , Physical Rev. A 1999, 59, 300.

[40] J. B. Pendry , J. Phys. C 1980, 13, 937.

[41] J. Schmidt , D. Hönig , P. Weckesser , F. Thielemann , T. Schaetz , L. Karpa , Appl. Phys. B 2020, 126, 176, DOI: 10.1007/s00340-020-07491-8. 33088025 PMC7547030

[42] A. R. Milosavljević, A. Giuliani, C. Nicolas, Gas-Phase Near-Edge X-Ray Absorption Fine Structure (NEXAFS) Spectroscopy of Nanoparticles, Biopolymers, and Ionic Species, in *X-ray and Neutron Techniques for Nanomaterials Characterization*, pages 451–505, Springer Berlin Heidelberg, Berlin, Heidelberg **2016**.

[43] M. F. Peintinger , D. V. Oliveira , T. Bredow , J. Comput. Chem. 2013, 34, 451. 23115105 10.1002/jcc.23153

[44] A. D. Becke , Phys. Rev. A 1988, 38, 3098. 10.1103/physreva.38.3098 9900728

[45] E. van Lenthe , J. G. Snijders , E. J. Baerends , J. Chem. Phys. 1996, 105, 6505.

[46] F. Neese , F. Wennmohs , U. Becker , C. Riplinger , J. Chem. Phys. 2020, 152, 224108, DOI: 10.1063/5.0004608. 32534543

[47] M. W. Buckley , N. A. Besley , Chem. Phys. Lett. 2011, 501, 540.

[48] P. Thompson , D. E. Cox , J. B. Hastings , J. Appl. Crystallogr. 1987, 20, 79.

[49] Y. Imamura , H. Nakai , Chem. Phys. Lett. 2006, 419, 297.

