# Supplementary material for: Structures of Gas‐Phase Hydrated Phosphotyrosine Revealed by Soft X‐ray Action Spectroscopy
Source: Chemistry. 2025 Jan 21;31(10):e202403665. doi: 10.1002/chem.202403665 (PMC11833222; doi:10.1002/chem.202403665)
Supplement: Supplementary file 1 — Supporting Information [file CHEM-31-e202403665-s001.pdf]

Chemistry–A European Journal

Supporting Information

Structures of Gas-Phase Hydrated Phosphotyrosine Revealed by Soft X-ray Action Spectroscopy

Juliette Leroux,* Jean-Yves Chesnel, Carlos Ortiz-Mahecha, Aarathi Nair, Bart Oostenrijk, Laura Pille, Florian Trinter, Lucas Schwob, and Sadia Bari*

Structures of Gas-Phase Hydrated Phosphotyrosine Revealed by Soft X-ray Action Spectroscopy: Supporting Information

Juliette Leroux,*[a][b] Jean-Yves Chesnel,[a] Carlos Ortiz-Mahecha,[c] Aarathi Nair,[d] Bart Oostenrijk,[b] Laura Pille,[b] Florian Trinter,[e] Lucas Schwob,[b] and Sadia Bari*[b][f]

[a] CIMAP, CEA/CNRS/ENSICAEN/Université de Caen Normandie, 14050 Caen, France.

[b] Deutsches Elektronen-Synchrotron DESY, 22603 Hamburg, Germany.

[c] Hamburg University of Technology, 21073 Hamburg, Germany.

[d] The Hamburg Centre for Ultrafast Imaging, Hamburg, Germany.

[e] Molecular Physics, Fritz-Haber-Institut der Max-Planck-Gesellschaft, 14195 Berlin, Germany.

[f] Zernike Institute for Advanced Materials, University of Groningen, 9747 AG Groningen, The Netherlands.

1 Methods

1.1 Experimental methods

The setup relies on a high-fluence electrospray ionization (ESI) source to introduce the ions into the gas phase. The electrosprayed ions are transported by a capillary into the first pumping stage, which hosts two skimmers that focus the ions and play the role of conductance limit between the chambers. The ions continue through a radio frequency (RF) octupole ion guide to a quadrupole mass filter (QMS) where the ions of interest are selected according to their mass-to-charge (m/z) ratio. Then, a second RF octupole and a set of Einzel lenses guide and focus the ions into an RF 3D ion trap, where they are accumulated before irradiation with photons. To allow efficient trapping of the ions, their kinetic energy is cooled down to room temperature by collisions with a helium buffer gas. After irradiation with X-ray photons for 2 seconds, all cationic products are extracted from the trap and analyzed in a reflectron time-of-flight (Re-TOF) mass spectrometer ($\mathrm{m}/\Delta\mathrm{m} = 1800$). A sketch of the apparatus is shown in Figure S1.

At the carbon (C) K-edge, two sets of data were recorded: one over the range from 284 eV to 291 eV with 200 meV steps and an energy bandwidth of 250 meV and a second from 284.55 eV to 286.75 eV with 50 meV steps and an 81 meV bandwidth to improve the spectral resolution around the first resonance at 285 eV. At the oxygen (O) K-edge, the energy bandwidth was set to 80 meV, and the photon-energy scan was performed from 528 eV to 545 eV with steps of 0.1 eV. A photodiode placed downstream of the ion trap was used to record the photon flux.

To obtain a near-edge X-ray absorption mass spectroscopy (NEXAMS) spectrum, the areas under the peaks of all detected fragments in the photoinduced mass spectra were summed for each photon energy over the scanned energy ranges.

The NEXAMS spectra obtained were normalized to the photon flux and to the precursor ion intensity. To account for all sources of background ions and average over long-term fluctuations of the ESI source, the data acquisition was divided into repeated cycles of two mass scans. First, the mass spectrum resulting from the irradiation of the trapped molecules was recorded. For the second cycle, the ESI was switched off, and the mass spectrum resulting from the irradiation of the residual gas was taken and subtracted from the mass spectrum resulting from the irradiation of the trapped molecules. A spectrum without photons (ESI-only) was recorded to account for fragmentation prior to the irradiation.

The NEXAMS, or total ion yield (TIY) spectra, can be assumed to be good ap-

proximations of the X-ray absorption cross sections and, thus, comparable to the calculated DFT/ROCIS oscillator strengths. Indeed, the partial ion yields (PIY) for individual fragments are the results of the convolution of the absorption cross section of the molecule with the branching ratio of the different fragmentation pathways. By summing up the contribution of all fragments into a "total ion yield" spectrum, so-called NEXAMS spectrum, we approach the result of an absorption spectrum [1]. Nevertheless, disagreement in spectral intensity could arise when detecting multiple ions from the same photoabsorption event or due to discrepancies in the overall detection efficiency of ions. NEXAMS has been extensively used and compared with X-ray absorption calculations (density functional theory) as was used in this study [2]–[9].

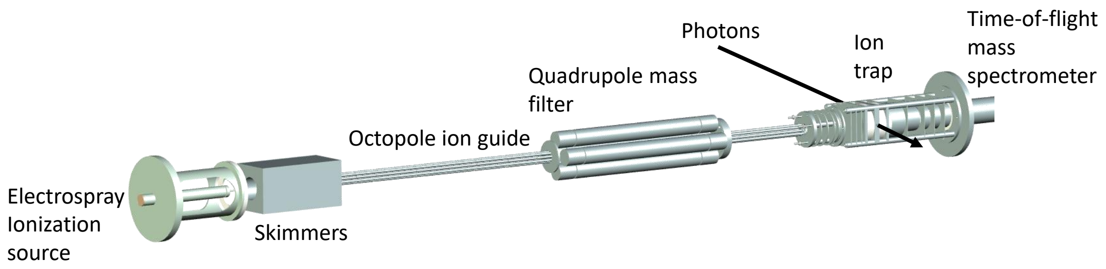

Figure S1: Scheme of the experimental setup used for measuring the NEXAMS spectra.

1.2 Theoretical methods

All calculations were performed using the combination of the density functional theory (DFT) and the restricted-open shell configuration interaction with singles (ROCIS) method, employing the TZVP Ahlrichs basis set [10] in combination with the B3LYP exchange-correlation functional [11]. Relativistic effects were considered using the zeroth order regular approximation (ZORA) [12]. All *ab initio* calculations have been performed using the ORCA electronic-structure package [13].

First, possible geometries of isolated protonated phosphotyrosine (pTyr) were calculated employing the DFT method. Twenty starting structures were generated from the PubChem database [14] and optimized using the Kohn-Sham ground-state determinant (DFT/ROCIS). All optimized conformers were then ranked according to their root mean square deviation (RMSD) values. The RMSD values with respect to each conformer have been calculated using the VMD software after alignment of every conformer with respect to their benzene ring to ensure proper calculation of the RMSD

value [15], [16]. It simply measures the average distance between the atoms of superimposed molecules. High RMSD values mean a large distance between the atoms, i.e., structures different from one another, whereas a low RMSD value means a small distance between the atoms and, thus, similar structures.

Finally, five structures were chosen for the isolated pTyr according to their final (lowest) DFT energies and RMSD values. The X-ray absorption spectra (XAS) at the C and O K-edges were obtained by calculating the density of states between the ground state and a set of excited states for these five structures and later compared with our experimental spectra.

To obtain the singly hydrated conformers of pTyr, initially, a solvation shell was created around the phosphate group of the five selected isolated structures to obtain hydrated structures. Subsequently, the water shell was narrowed down to five possible hydrated pTyr configurations for each isolated structure. For this, a solvation shell method was employed using Ambertools [17] integrated in the CHIMERA software [18]. The solvation process involved an 8.0 Å extended layer around the whole molecule using four-point TIP4P rigid water model [19] the CHIMERA software. The full solvation shell was further reduced to a 6 Å distance radius around the oxygen atoms of the phosphate group. The positions of the water molecules in the five initial systems were optimized with the semi-empirical quantum chemical method PM7 using the MOPAC software (the pTyr molecule was frozen during this calculation) [20].

To reduce the number of possibilities for the water-molecule positions from the full solvation shell of the five possible structures, we selected singly hydrated structures where the water molecule is either hydrogen-bonded to the phosphate group or bound to the NH_3^+, starting from the 6 Å shell around the phosphate group, or close to the benzene ring (on top of it). This gave four to six possible water-molecule positions for each selected conformer of pTyr. These possible structures were further optimized at the B3LYP/TZVP level of theory. The following categories were identified within the 24 hydrated conformers: 7 conformers have the water molecule making a bridge between the NH_3^+ group and the phosphate group (called NP_bridge in the following), and 15 of them have the water molecule making hydrogen bonds (HB) with the phosphate group only, called POH later in the text. The three lowest energy conformers of both categories were chosen as representatives, and their X-ray absorption spectra were calculated. Moreover, two hydrated conformers showed structures different from those of the 21 others. One had the water molecule making a single hydrogen bond with the NH_3^+ of the phosphotyrosine (Nterm in the following). The last conformer displayed a water bridge between the phosphate group and the carboxylic group of the phosphotyrosine, which is thus named the OP_bridge. Figure S2 shows the structures

of the eight conformers chosen for further analysis. The X-ray absorption spectra of the eight conformers were calculated at the carbon K-edge and compared with the experimental spectrum.

For that, the relative intensity of each resonance and the transition positions were considered to compare the calculated spectra with the experimental spectrum at the C K-edge. We employed the Pendry reliability factor R_p [21] for an unbiased criterion for this comparison. The equation used to calculate the R_p factor can be written as follows:

$$R_p = \frac{\sum_{i=0}^{n}(Y_1 - Y_2)^2}{\sum_{i=0}^{n}(Y_1^2 + Y_2^2)}, \tag{1.1}$$

with Y_i, i=1,2 being the renormalized logarithmic derivatives defined as

$$Y_i = \frac{L_i^{-1}}{(L_i^{-2} + V_0^2)},$$

with

$$L_i = \frac{\frac{\partial I_i}{\partial x}}{I_i}$$

being the derivative of the intensity as a function of the photon energy (x) over the intensity, and V_0 is the approximate half width of the peaks (here taken to be 0.6 eV). A perfect agreement between the theoretical spectrum and the experimental one is represented by $R_p = 0$, while $R_p = 1$ means no agreement.

Figure S3 shows the calculated spectra at the carbon K-edge for the chosen singly hydrated conformers and the bare one as well as their calculated R_p factor. Structures from which their calculated spectra at the carbon K-edge were not in good agreement with the experimental NEXAMS were discarded, i.e., POH_1, POH_3, NP_bridge_1, and NP_bridge_3. There is also the possibility that the water molecule binds only to the phosphate group. Therefore, POH_2, of which the XAS at the C K-edge fits the experimental spectrum, was considered a possible singly hydrated pTyr structure.

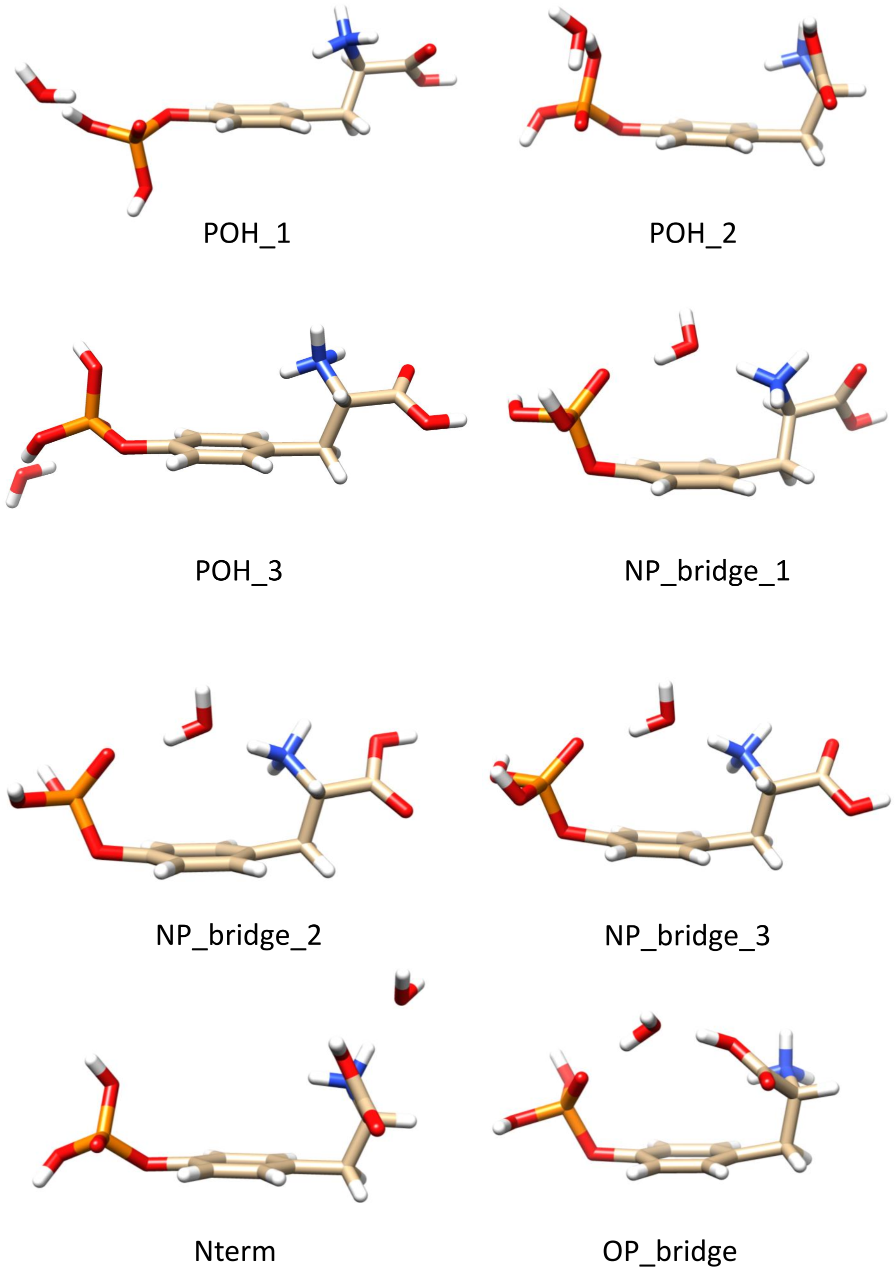

Figure S2: Structures of the eight singly hydrated conformers of pTyr.

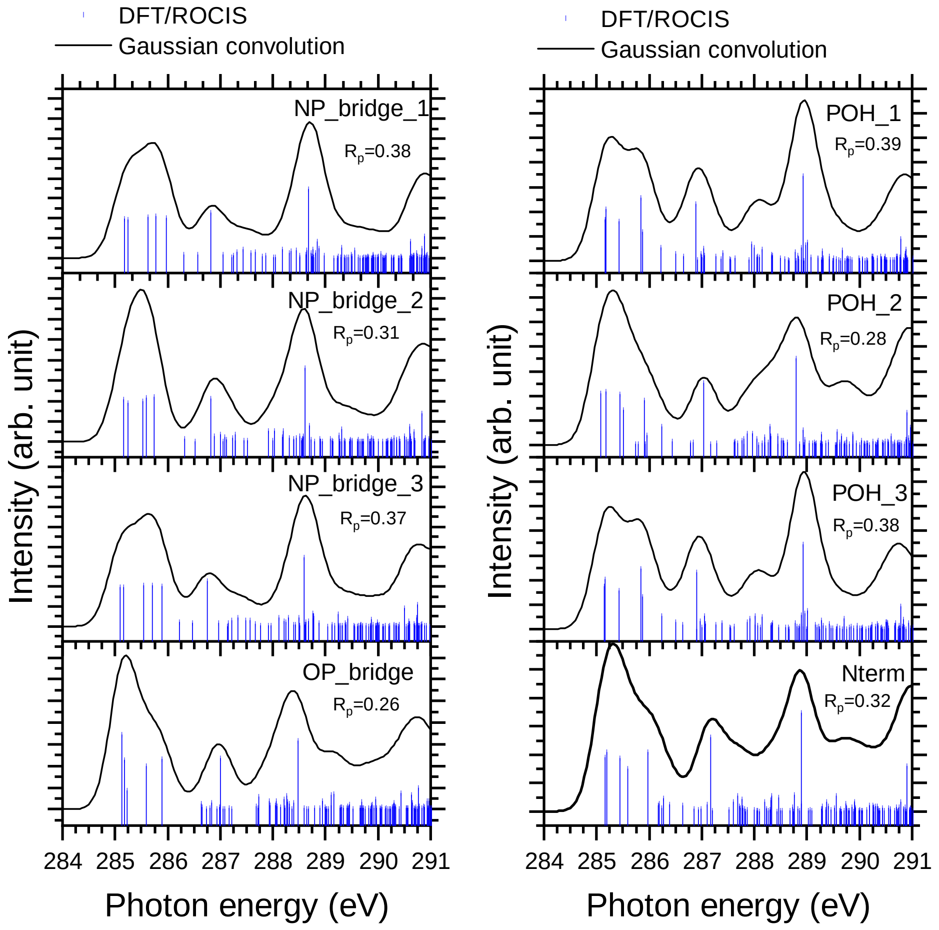

Figure S3: Simulated spectra at the C K-edge and their Pendry factors for all calculated hydrated conformers of pTyr. The absorption lines were shifted by approximately 11.5 eV.

2 Tables and Figures

Table S1: Attribution of the different fragments produced by the irradiation of $[pTyr+H+H_2O]^+$ and $[pTyr+H]^+$ with photons of 536.3 eV.

m/z	Attribution
47	$CO_2H_3{}^+$
50	PH_3O^+
51	$C_4H_3{}^+$
52	$C_4H_4{}^+$
62	$C_2O_2H_6{}^+$
63	$PO_2{}^+$
65	$C_5H_5{}^+$
78	$C_6H_6{}^+$
80	$C_6H_8{}^+$
81	$PO_3H_2{}^+$
99	$[PO_3H_2 + H_2O]^+$
107	$C_7H_7O^+$
108	$C_7H_8O^+$

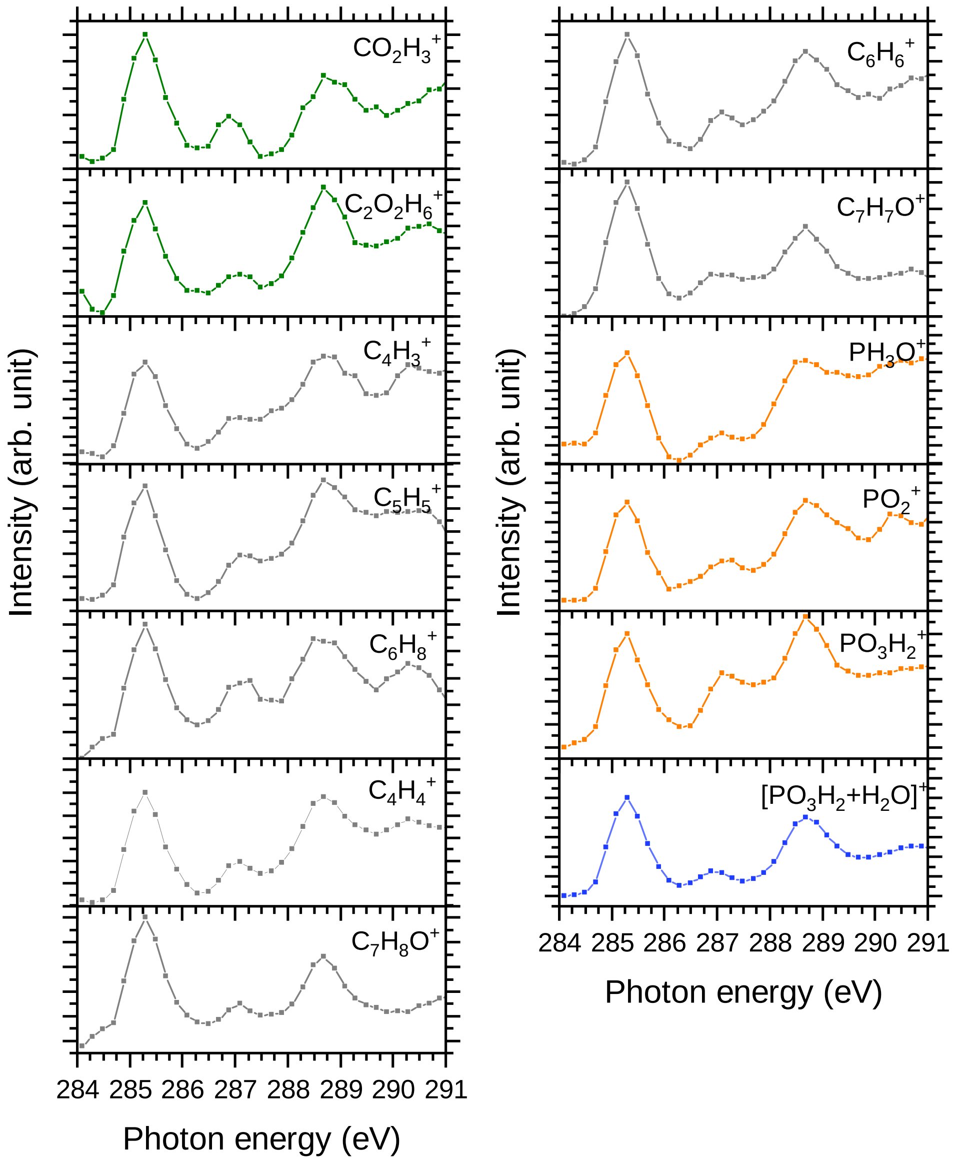

Figure S4: PIYs of the different fragments originating from the mixture of $[pTyr+H+H_2O]^+$ and $[pTyr+H]^+$ at the C K-edge. All PIYs have been normalized on the resonances at 285.2 eV. The PIYs of the carboxyl-containing fragments are shown in green, and the tyrosine side-chain fragments are shown in grey. The PIYs of the phosphorus-containing fragments are shown in orange, and the PIY of the hydrated fragment is shown in blue.

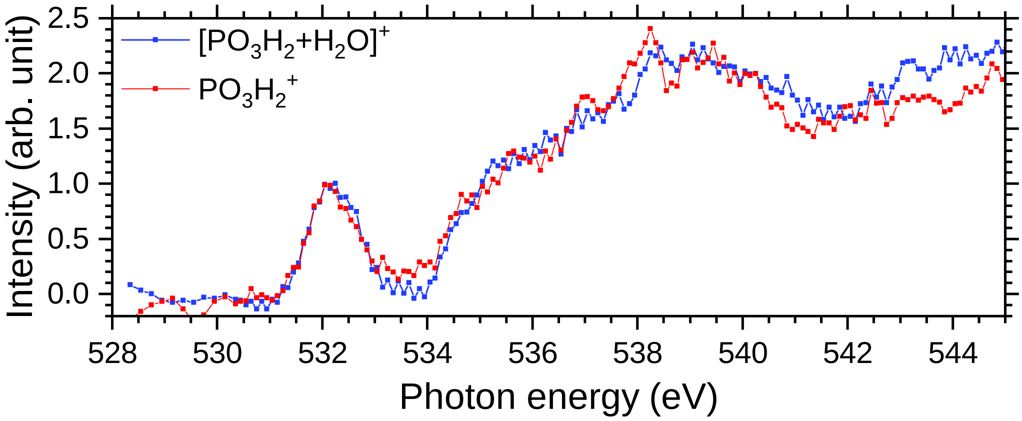

Figure S5: Ion yield spectra of the hydrated and isolated PO_3H_2 fragments. Both spectra have been baseline-corrected at 529 eV and normalized on the π*(C=O) resonance at 532.15 eV.

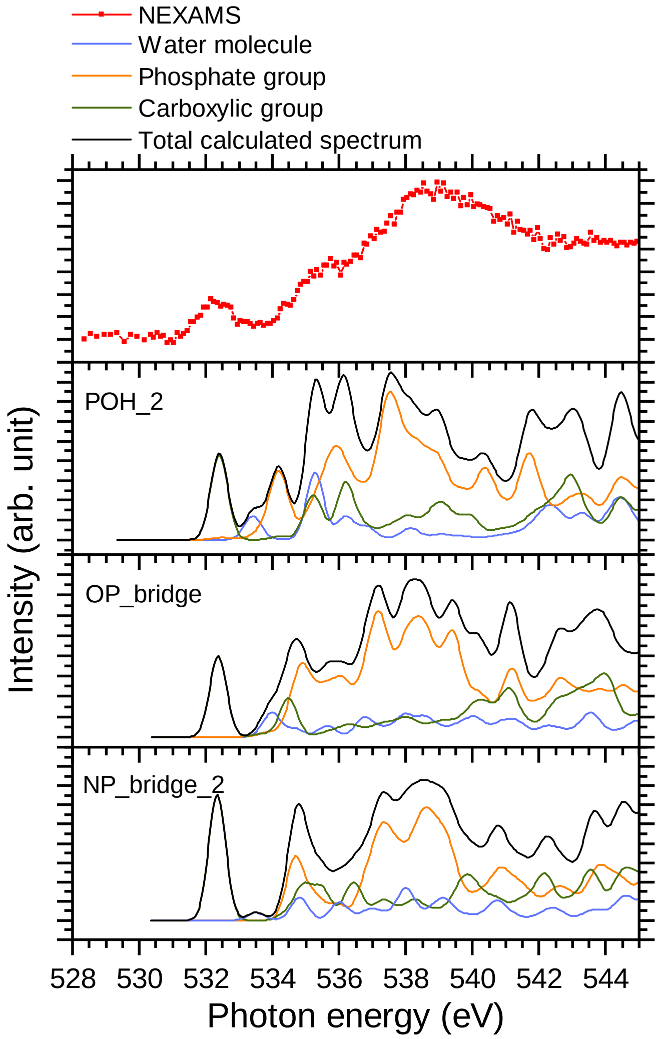

Figure S6: Comparison of the experimental spectrum at the O K-edge with the decomposition of the calculated spectra for the three singly hydrated conformers. The orange curve represents the contribution of the oxygen atoms of the phosphate group to the calculated spectra. The contribution of the oxygen atoms of the carboxylic group is represented in brown, and the oxygen atom of the water molecule is represented in blue. The convolution of the theoretical transitions has been done with a sum of Gaussian functions of FWHM = 0.6 eV.

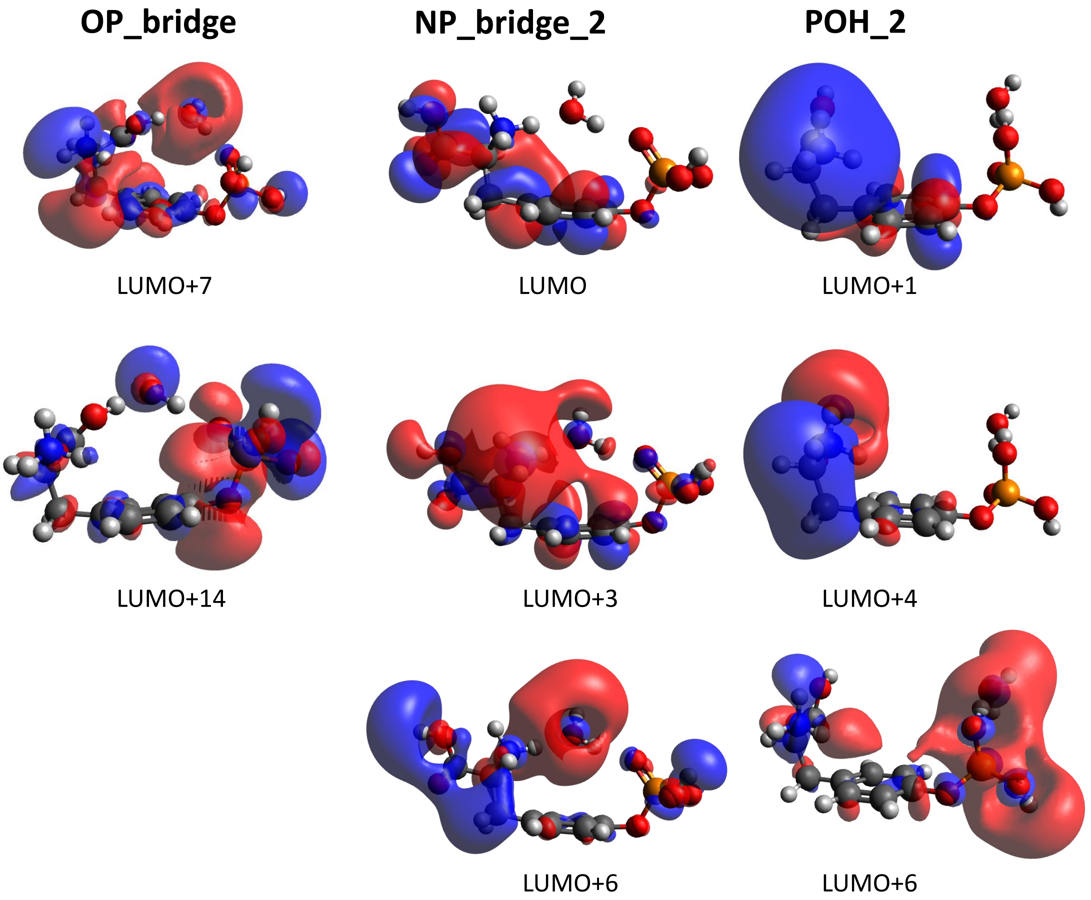

Figure S7: Molecular orbitals responsible for the main transitions at the oxygen K-edge for the OP_bridge, NP_bridge_2, and POH_2 conformers.

References

[1] A. R. Milosavljević, A. Giuliani, and C. Nicolas, "Gas-Phase Near-Edge X-Ray Absorption Fine Structure (NEXAFS) Spectroscopy of Nanoparticles, Biopolymers, and Ionic Species," in *X-ray and Neutron Techniques for Nanomaterials Characterization*, Berlin, Heidelberg: Springer Berlin Heidelberg, 2016, pp. 451–505, ISBN: 9783662486061. DOI: 10.1007/978-3-662-48606-1{_}8.

[2] L. Schwob, S. Dörner, K. Atak, *et al.*, "Site-Selective Dissociation upon Sulfur L-Edge X-ray Absorption in a Gas-Phase Protonated Peptide," *Journal of Physical Chemistry Letters*, vol. 11, no. 4, pp. 1215–1221, Feb. 2020, ISSN: 19487185. DOI: 10.1021/acs.jpclett.0c00041.

[3] G. Mattioli, R. Schürmann, C. Nicolafrancesco, A. Giuliani, and A. R. Milosavljević, "Effect of Protonation on the Molecular Structure of Adenosine 5-Triphosphate: A Combined Theoretical and Near Edge X-ray Absorption Fine Structure Study," *Journal of Physical Chemistry Letters*, vol. 14, no. 45, pp. 10 173–10 180, 2023, ISSN: 19487185. DOI: 10.1021/acs.jpclett.3c01666.

[4] X. Wang, S. Rathnachalam, K. Bijlsma, *et al.*, "Site-selective soft X-ray absorption as a tool to study protonation and electronic structure of gas-phase DNA," *Physical Chemistry Chemical Physics*, vol. 23, no. 20, pp. 11 900–11 906, May 2021, ISSN: 14639076. DOI: 10.1039/d1cp01014j.

[5] K. Schubert, M. Guo, K. Atak, *et al.*, "The electronic structure and deexcitation pathways of an isolated metalloporphyrin ion resolved by metal L-edge spectroscopy," *Chemical Science*, vol. 12, no. 11, pp. 3966–3976, Mar. 2021, ISSN: 20416539. DOI: 10.1039/d0sc06591a.

[6] S. Dörner, L. Schwob, K. Atak, *et al.*, "Probing Structural Information of Gas-Phase Peptides by Near-Edge X-ray Absorption Mass Spectrometry," *Journal of the American Society for Mass Spectrometry*, vol. 32, no. 3, pp. 670–684, Mar. 2021, ISSN: 18791123. DOI: 10.1021/jasms.0c00390.

[7] G. Gopakumar, P. H. Svensson, O. Grånäs, *et al.*, "X-ray Induced Fragmentation of Protonated Cystine," *Journal of Physical Chemistry A*, vol. 126, no. 9, pp. 1496–1503, 2022, ISSN: 15205215. DOI: 10.1021/acs.jpca.1c10158.

[8] Y. J. Chiang, W. C. Huang, C. K. Ni, C. L. Liu, C. C. Tsai, and W. P. Hu, "NEXAFS spectra and specific dissociation of oligo-peptide model molecules," *AIP Advances*, vol. 9, no. 8, Aug. 2019, ISSN: 21583226. DOI: 10.1063/1.5112151.

[9] I. Ljubić, A. Kivimäki, and M. Coreno, "An experimental NEXAFS and computational TDDFT and ΔdFT study of the gas-phase core excitation spectra of nitroxide free radical TEMPO and its analogues," *Physical Chemistry Chemical Physics*, vol. 18, no. 15, pp. 10 207–10 217, 2016, ISSN: 14639076. DOI: 10.1039/c6cp00490c.

[10] M. F. Peintinger, D. V. Oliveira, and T. Bredow, "Consistent Gaussian basis sets of triple-zeta valence with polarization quality for solid-state calculations," *Journal of Computational Chemistry*, vol. 34, no. 6, pp. 451–459, 2013, ISSN: 01928651. DOI: 10.1002/jcc.23153.

[11] A. D. Becke, "Density-functional exchange-energy approximation with correct asymptotic behavior," *Physical Review A*, vol. 38, no. 6, pp. 3098–3100, Sep. 1988, ISSN: 0556-2791. DOI: 10.1103/PhysRevA.38.3098.

[12] E. Van Lenthe, J. G. Snijders, and E. J. Baerends, "The zero-order regular approximation for relativistic effects: The effect of spin-orbit coupling in closed shell molecules," *Journal of Chemical Physics*, vol. 105, no. 15, pp. 6505–6516, 1996, ISSN: 00219606. DOI: 10.1063/1.472460.

[13] F. Neese, F. Wennmohs, U. Becker, and C. Riplinger, "The ORCA quantum chemistry program package," *The Journal of Chemical Physics*, vol. 152, no. 22, p. 224 108, Jun. 2020, ISSN: 0021-9606. DOI: 10.1063/5.0004608.

[14] S. Kim, E. E. Bolton, and S. H. Bryant, "PubChem3D: conformer ensemble accuracy," *Journal of Cheminformatics*, vol. 5, no. 1, p. 1, Dec. 2013, ISSN: 1758-2946. DOI: 10.1186/1758-2946-5-1.

[15] W. Humphrey, A. Dalke, and K. Schulten, "VMD: Visual molecular dynamics," *Journal of Molecular Graphics*, vol. 14, no. 1, pp. 33–38, Feb. 1996, ISSN: 02637855. DOI: 10.1016/0263-7855(96)00018-5.

[16] J. Eargle, D. Wright, and Z. Luthey-Schulten, "Multiple Alignment of protein structures and sequences for VMD," *Bioinformatics*, vol. 22, no. 4, pp. 504–506, Feb. 2006, ISSN: 1367-4811. DOI: 10.1093/bioinformatics/bti825.

[17] D. A. Case, T. E. Cheatham III, T. Darden, *et al.*, "The amber biomolecular simulation programs," *Journal of Computational Chemistry*, vol. 26, no. 16, pp. 1668–1688, 2005. DOI: https://doi.org/10.1002/jcc.20290.

[18] E. F. Pettersen, T. D. Goddard, C. C. Huang, *et al.*, "UCSF Chimera - A visualization system for exploratory research and analysis," *Journal of Computational Chemistry*, vol. 25, no. 13, pp. 1605–1612, 2004, ISSN: 01928651. DOI: 10.1002/jcc.20084.

[19] E. Sanz, C. Vega, J. L. F. Abascal, and L. G. MacDowell, "Phase diagram of water from computer simulation," *Phys. Rev. Lett.*, vol. 92, p. 255 701, 25 Jun. 2004. DOI: 10.1103/PhysRevLett.92.255701.

[20] J. J. Stewart, "MOPAC: A semiempirical molecular orbital program," *Journal of Computer-Aided Molecular Design*, vol. 4, no. 1, pp. 1–103, 1990, ISSN: 0920654X. DOI: 10.1007/BF00128336.

[21] J. B. Pendry, "Reliability factors for LEED calculations," *Journal of Physics C: Solid State Physics*, vol. 13, no. 5, pp. 937–944, Feb. 1980, ISSN: 0022-3719. DOI: 10.1088/0022-3719/13/5/024.
